# Pharmacy-based interventions to increase vaccine uptake: report of a multidisciplinary stakeholders meeting

**DOI:** 10.1186/s12889-019-8044-y

**Published:** 2019-12-18

**Authors:** Fiona Ecarnot, Gaetano Crepaldi, Philippe Juvin, John Grabenstein, Giuseppe Del Giudice, Litjen Tan, Susan O’Dwyer, Susanna Esposito, Xavier Bosch, Gaetan Gavazzi, John Papastergiou, Jacques Gaillat, Robert Johnson, Marco Fonzo, Andrea Rossanese, Caterina Suitner, Jane Barratt, Alberta di Pasquale, Stefania Maggi, Jean-Pierre Michel

**Affiliations:** 1 EA3920, University of Franche-Comté, Besancon, France; 2 Department of Cardiology, University Hospital Jean Minjoz, Boulevard Fleming, 25000 Besancon, France; 3 University of Padua, Padua, Italy; 4 Global Product Quality Management, Sanofi Pasteur, Lyon, France; 5 Executive Director, Global Vaccines Medical Affairs, Merck Research Laboratories, North Wales, PA 19454 USA; 6 Translational Science Leader, R&D Center Italy, GSK Vaccines Srl, 53100 Siena, Italy; 7 Chief Strategy Officer, Immunization Action Coalition, United States; Co-Chair, National Adult and Influenza Immunization Summit, Saint Paul, MN, USA; 8 Boots Retail (Ireland) Limited, Nangor Road, Clondalkin, Dublin, 12 Ireland; 9 Pediatric Clinic, Department of Biomedical and Surgical Sciences, Università degli Studi di Perugia, Perugia, Italy; 10 Catalan Institute of Oncology, Cancer Epidemiology Research Program, L’Hospitalet de Llobregat (Barcelona), Barcelona, Spain; 11 University of Grenoble-Alpes, GREPI, FRE 3405 CNRS and University Clinic of Geriatric Medicine, University hospital of Grenoble, Grenoble, France; 12 Leslie Dan Faculty of Pharmacy, University of Toronto, Toronto, Canada; 13 Centre Hospitalier Annecy-Genevois, Annecy, France; 14 Hon. Senior Research Fellow, Faculty of Health Sciences: Emeritus Consultant in Pain Medicine, University of Bristol, Bristol, UK; 15 DCTV - Department of Cardiac, Thoracic and Vascular Sciences, Hygiene and Public Health Unit, University of Padua, Padua, Italy; 16 Centre for Tropical Diseases, “Sacro Cuore - Don Calabria” Hospital, Negrar, Verona, Italy; 17 Department of Developmental and Social Psychology, University of Padova, Padova, Italy; 18 International Federation on Ageing, Toronto, Ontario Canada; 19 GSK, Wavre, Belgium; 20 CNR, Institute of Neuroscience – Aging Branch, Padua, Italy; 21 University of Geneva, Geneva, Switzerland

**Keywords:** Vaccination, Pharmacists, Pharmacy-based interventions, Vaccine coverage

## Abstract

**Background:**

Despite the existence of efficacious vaccines, the burden of vaccine-preventable diseases remains high and the potential health benefits of paediatric, adolescent and adult vaccination are not being achieved due to suboptimal vaccine coverage rates.

**Main body of abstract:**

Based on emerging evidence that pharmacy-based vaccine interventions are feasible and effective, the European Interdisciplinary Council for Ageing (EICA) brought together stakeholders from the medical and pharmacy professions, the pharmaceutical industry, patient/ageing organisations and health authorities to consider the potential for pharmacy-based interventions to increase vaccine uptake. We report here the proceedings of this 3-day meeting held in March 2018 in San Servolo island, Venice, Italy, focussing firstly on examples from countries that have introduced pharmacy-based vaccination programmes, and secondly, listing the barriers and solutions proposed by the discussion groups.

**Conclusions:**

A range of barriers to vaccine uptake have been identified, affecting all target groups, and in various countries and healthcare settings. Ease of accessibility is a potentially modifiable determinant in vaccine uptake, and thus, improving the diversity of settings where vaccines can be provided to adults, for example by enabling community pharmacists to vaccinate, may increase the number of available opportunities for vaccination.

## Introduction

The incidence of vaccine-preventable diseases has decreased dramatically in recent decades. The incidence of influenza (flu), pneumococcal disease and herpes zoster has been substantially reduced thanks to vaccination [1, 2]. The potential health benefits and cost savings that could be yielded from vaccination are not being achieved due to suboptimal vaccine coverage rates in most European countries.

A range of barriers to vaccine uptake have been identified [3–6]. Ease of accessibility is a potentially modifiable determinant, and thus, improving the diversity of settings where vaccines can be provided to adults will likely increase the number of available opportunities for vaccination, thus improving vaccine-coverage rates [7]. One possible setting where adult vaccines can be given is in the community pharmacy. The role played by community pharmacies varies widely around the world. Pharmacies remain a privileged point of contact with the public, and face challenges in managing patients and providing optimum service, while also wrestling with issues such as medication supply, storage, and handling. Pharmacists are often the most frequently consulted healthcare professional for many individuals, and are often consulted for healthcare advice. Clearly, the pharmacist’s role is polyvalent, and their pharmacies are an ideal point of contact with the potential to implement positive healthcare initiatives. To fulfil the key role that they could play in vaccination, it is important that pharmacists are well informed about vaccine research and development, product characteristics as well as manufacturing specificities and complexity.

## Main text

In this context, the European Interdisciplinary Council for Ageing (EICA) has an important role to play in fostering interdisciplinary analyses, initiating high-level discussions, as well as in translating and disseminating the results of ageing research to various professional groups, policy makers, and the general public. Based on emerging evidence that pharmacy-based vaccine interventions are feasible and effective, the EICA sought to bring together stakeholders from the medical and pharmacy professions, the pharmaceutical industry, patient/ageing organisations and health authorities to consider the potential for pharmacy-based interventions to increase vaccine uptake. To this end, a 3-day meeting was organized from 14 to 16 March 2018, on San Servolo Island, Venice, Italy, bringing together almost 100 international participants (75% from the public sector (pharmacies, professional societies, universities) and 25% from the private sector (pharmaceutical industry)).

We report here the proceedings of this meeting, focussing firstly on examples from countries that have introduced pharmacy-based vaccination programmes, and secondly, listing the barriers and solutions proposed by the discussion groups. This report supplements a recent survey from the International Pharmaceutical Federation of the activity of pharmacists as immunizers in multiple countries [8].

### Examples of countries that have introduced pharmacy-based vaccine services

#### The Republic of Ireland

Ireland is an island nation situated on the western edge of the European Union, with a population of around 4.76 million. As of early 2018, there were 3625 community pharmacists working in 1849 community pharmacies [9], representing around 37.5 pharmacies per 100,000 inhabitants, which is slightly higher than the OECD average of 24.7/100,000 [10]. In 2008, the Pharmaceutical Society of Ireland (PSI) published an interim report on how the pharmacy profession could contribute to the development of a more integrated approach to healthcare in Ireland in order to enhance services to patients. The report concluded that pharmacists could make a significant impact in critical areas of healthcare, for several reasons. Firstly, pharmacies are the primary care service with the greatest reach into the general population, and their typically long opening hours provide ease of accessibility. Secondly, pharmacists have the most frequent contact with the population in Ireland. Thirdly, the pharmacist is ideally placed to assess, identify, contact and encourage at-risk individuals in the population as a whole and initiate an intervention that would result in vaccine uptake. With these factors in mind, the report called for the development of a national policy and strategy for maximising the use of vaccines, taking account of the huge potential of the community pharmacy network, to improve patient outcomes and take pressure off other healthcare providers. It also called for the development of national standards and protocols for the delivery of vaccination and immunisation programmes through community pharmacies in Ireland.

In 2011, a change to the legislation in Ireland introduced new provisions allowing for pharmacist-led influenza vaccination [11], expanding in 2015 to include herpes zoster and pneumococcal vaccination [12]. The PSI, the pharmacy regulator in Ireland, published guidance on the provision of vaccination services by pharmacists in the retail pharmacy setting [13]. In partnership with the Irish Institute of Pharmacy, the PSI developed a modular system of training to equip pharmacists with the necessary skills and knowledge to safely administer medicines and vaccines to patients in the retail pharmacy setting. Pharmacy-based vaccination was rolled out starting with the seasonal influenza vaccination in the 2011/2012 season, when a total of 9125 vaccines were delivered by pharmacists, rapidly increasing to almost 80,000 vaccines by the 2016/2017 season in Ireland. The PSI conducts audits and inspections to ensure the services are being delivered to the correct standards. In the most recent evaluation of this service, patient satisfaction with this service was high, with 95% of patients declaring that they were very satisfied with the information received from the pharmacist about vaccination, and 93% rating overall flu service at 9 or 10 out of 10 [14]. This report noted that 47% of patients indicated that their motivation for attending the pharmacy for the flu vaccination was the convenience this service offered, combined with the shorter waiting times (cited by 28%), while cost was a motivating factor for only 13% of patients, likely because many patients receive the vaccines for free under the national healthcare system [14].

Several challenges beset the path to pharmacy-based vaccination in Ireland, including storage requirements, difficulties meeting vaccine delivery schedules, administrative issues including funding and compatibility between data recording systems, inter-professional communication, workload management and vaccine hesitancy among the public. Once these barriers were overcome, and pharmacy-based vaccination was successfully launched, progress was then made in expanding the portfolio of available vaccines, and there is likely potential for further developments in the future. Some adaptations may be necessary according to the different vaccines proposed, for example due to differences in administration schedules. However, the overall success of the Irish experience in introducing pharmacy-based vaccination services underlines the feasibility of this model of healthcare delivery, and can serve as a model for other countries who are considering doing the same.

#### Canada

Canadians experience long wait times for access to healthcare services [15] and this is a major public health challenge. By diverting some of the care to pharmacies, wait times can be significantly reduced. Public trust in the community pharmacist is high in Canada, and pharmacists have the competency and skills required to fully embrace enhanced scope of practice. Since 2005, the pharmacists’ scope of practice has been substantially expanded in Canada, at varying rates across the different provinces. With respect to vaccination, pharmacists are authorized to inject flu vaccines, and in some provinces, also other vaccines funded by public or private payers. Reimbursement models vary by jurisdiction and are major determinants for the successful implementation of vaccination programmes. Many pharmacies are reluctant to adopt these services in the absence of appropriate funding. Flu and other public vaccines that are refunded by the public health system are generally adopted on a wide scale. Indeed, in 2016 in Canada, 2.2 million injections were given in pharmacies, of which 93% were flu vaccines. As noted by patient feedback in the Republic of Ireland, convenience and accessibility are major drivers of success. Other advantages include cost savings for the healthcare system, reduced physician workload and wait times, and overall economic improvement. It is noteworthy that the number of vaccines given is increasing, but physician vaccination is not decreasing noticeably, which underlines the fact that pharmacy-based vaccine services target different individuals who may not be reached by the existing system via family doctors or other primary care centres. This debunks the argument that pharmacy-based vaccine services are in competition with general practitioners (GPs) and primary care. In addition, vaccination services provide an additional source of revenue for pharmacists.

The body of evidence evaluating the impact of pharmacy-based vaccination in Canada is growing. In a study comparing estimated influenza vaccine coverage before and after pharmacists began administering publicly funded influenza immunizations in Nova Scotia, Isenor et al. reported that in 2013–2014, the vaccination coverage rate increased to 42%, up from 36% in 2012–2013 (*p* < 0.001), and pharmacists administered over 78,000 influenza vaccinations overall, corresponding to nearly 9% of the province’s population over the age of five [16]. Influenza vaccine coverage rates for those aged 65 years and older also increased by 9.8% (p < 0.001) in 2013–2014, compared to 2012–2013. By comparison, pooled data from the 2007–2014 cycles of the Canadian Community Health Survey (*n* = 481,526) showed that the introduction of a policy for pharmacist administration of influenza vaccine was associated with a modest increase in coverage (2.2%) and an individual’s likelihood of uptake (adjusted prevalence ratio 1.05, 95% confidence interval 1.02–1.08) [17]. Taken together, these data provide robust evidence that pharmacy-based vaccination programmes improve vaccine uptake in the community, and highlight again the feasibility of rolling out such services, even in geographical areas as large as Canada, despite differing regulations and rates of legislative change across the various provinces.

#### Contrasting evidence

In contrast to the Canadian experience, there has been some conflicting evidence from the United Kingdom (UK) regarding the usefulness of pharmacist vaccination. Two studies evaluating community pharmacy based vaccination services reported high patient satisfaction and improved convenience, but no impact on vaccine uptake and only weak evidence that the system widens access to previously unvaccinated subjects [18, 19]. In an online survey of London pharmacists delivering vaccines, Atkins et al. reported that there was potential for improved convenience, but no statistically significant increase in uptake over the period studied, although vaccine delivery in the pharmacy cost less than administration by a GP [20]. These results should be interpreted in light of the regulatory environment surrounding pharmacy-based vaccine administration in the UK, with the simultaneous use of two reporting systems to gather data about vaccine delivery, as well as voluntary participation in the programmes. However, they do suggest that programmes to introduced pharmacy-based vaccine administration may not meet with the same success everywhere, and will likely require adaptation to account for local conditions, regulatory environment and pre-existing services.

### Strategies towards overcoming barriers

A wide range of barriers exist to increased vaccine uptake via pharmacy-based interventions, including regulatory, professional, personal (at the level of pharmacists individually and as a profession), and financial obstacles. During our meeting, focus groups identified the main barriers to the implementation of pharmacy-based vaccine services, and proposed solutions for overcoming them.

#### Engage the profession

The first important strategy is to increase the involvement of pharmacies in vaccine implementation by motivating pharmacists, as a profession, to engage in the activity. They must come together as a single body to voice their support and commitment to changing the social context of pharmacists vaccinating, with this new service introduced, approved and widely used. This unified approach from the profession as a whole, represented by its professional organisations, guilds or other bodies, will be necessary to provide the momentum and political will to overcome the barriers, particularly a need for a change to existing legislation.

#### Training

It is important to develop the necessary competence within the profession to ensure that if the lobbying is successful, then the pharmacists will be sufficient in number, and adequately trained to implement the service and manage any complications. This calls for professional bodies to define suitable learning objectives in line with the services planned to be implemented. This includes but is not limited to assessing the person’s vaccination status, advising on appropriate vaccinations considering the person’s age and health status, prescribing and/or dispensing vaccines, administering vaccines, monitoring for complications, dealing with any emergency complications that may occur, and notifying and reporting adverse events. Such curricula have been developed by pharmacists in multiple countries. Then, a training programme for the profession must be developed, so that a pharmacist who has successfully completed the training can be considered to have the skills and competences required to implement the planned service. This may require endorsement from one or several universities, or faculties, to deliver an academic diploma commensurate with the level of training, thus giving credence to the level of qualification. Nationwide rolling out of the training must be implemented to ensure that all pharmacies have at least one qualified individual available to perform vaccination when the service becomes available to the public. Training and qualifications already exist in a number of countries; in the United States of America, for example, the American Pharmacists Association provides certified training that is now used in other regions as well, and the International Pharmaceutical Federation (FIP) offers this training to its members.

#### Advocacy and lobbying

Once the professionals are motivated as a single voice, and have acquired the necessary skills, advocacy and lobbying are the next steps. The regulatory situation of the country must be assessed to identify which dispositions of the law or regulation need to be changed or adapted, in terms of the description of scope and the modalities of practice, to allow the service to be implemented. This effort can often proceed simultaneously with the previous two points. New requirements may need to be laid down if no such services were ever provided in pharmacies, in the interests of public health, such as technical details on the ordering, storage and dispensation of vaccine products, management of staff and vaccination schedules, minimal facilities to ensure patient privacy on the premises, etc. These requirements need to be enshrined in the law and/or regulation, with guidelines for practical implementation by the profession. The discussions surrounding the exact modalities for implementing vaccine strategies in pharmacies should ideally take place in open dialogue with other healthcare professionals and other interested stakeholders. The objective is complementarity between healthcare professionals, for the benefit of the population, by improving access to adult vaccination, thus expanding possibilities to reach a broader audience and ensure wider availability of vaccines for the public.

#### Internal regulatory diversity

In countries that have a federal governmental structure, such as Canada or Switzerland, or other forms of regional decentralization of healthcare provision, internal regulatory diversity is an added challenge, since the laws or regulations then need to be changed in each individual province, state or canton. In addition, the scope given to pharmacists in terms of vaccination implementation may differ between jurisdictional entities. Nonetheless, evidence shows that when one or several regions successfully initiate the service and collect evidence in support of its efficacy, then through a domino effect, the other regions often follow suit, as reported by studies of the Canadian experience [16, 17] .

#### Generating evidence of efficacy

Generating evidence of the efficacy of pharmacy-based implementation of vaccine strategies is important to maintain motivation among the profession, and provide a rationale that policy-makers can use to justify wider implementation. Documenting is vital to establishing a precedent for other regions within the same countries, or for other countries to follow. To create such data, it is ideal to start with a small group of motivated, engaged pharmacists, and work in collaboration with academics or other partners to assess implementation and effectiveness using validated performance measures. This will help to generate robust evidence of expanding coverage, increased patient satisfaction, and proof that pharmacy-based vaccination reaches new populations who now get a vaccine where they never did before, as a result of improved access to vaccines. Evidence will consolidate the service and support advocacy for third party remuneration at a regional and national level, which is important for sustainability.

#### Access to health records

Access to health records is an important element of strategy, not only for vaccination but for most pharmacy services in general to provide better service. It is essential to have access to reliable data about what vaccines the patient already received, to enable the pharmacist to advise appropriately, and take measures by providing vaccination or referring the patient to another provider. Self-reported vaccination history is not reliable enough for pharmacists to administer vaccines on this basis alone. However, in the absence of universal electronic medical health records, the issue of medical data is thorny. In particular, such questions as who hosts the information, who can view it, who can modify it (by adding or deleting), and whether and how it can include payment data, are dealt with differently, if at all, within and between countries. Overall, an immunization registry system that is complete, yet flexible enough for all stakeholders involved (healthcare professionals, patients, payers) remains to be identified. Countries with vaccinating pharmacists have adopted various solutions [8].

#### Funding

As with all public health policies, the question of funding is vital to ensuring the sustainability of pharmacy-based vaccine interventions. The administration of vaccines undeniably represents an extra workload for pharmacists, and adequate compensation should be provided to all immunizers. Some vaccines may be covered by national health systems, while others are not, and this may influence the choice of the vaccines that pharmacists are willing to provide. Ideally, reimbursement for pharmacy immunization should be integrated into the national health system (if one exists) in the same way as for drugs that are dispensed there. This is a point that needs to be negotiated in every country, region or canton between relevant stakeholders, according to national structures for public and private healthcare coverage [8].

#### International collaboration for advocacy

At an international level, there is a compelling need for a joint action plan on national vaccination policies. Despite the existence of European Union recommendations, most countries organise vaccine delivery differently, and a joint action plan at an international level would facilitate implementation of vaccination programmes in a more uniform manner. Advocacy should also include the necessity to have different pharmacy and medical societies engage in dialogue, to reach a consensus on how to improve coverage in each country by defining their respective roles and responsibility. Concerted collaborative action at an international level to improve access to adult vaccinations may also send a strong message to the public, and help reduce vaccine hesitancy and strengthen the supply of vaccines. To move towards this goal, professional organisations need to call on governments to involve pharmacies in vaccination, and lobby policy-makers to change legislation in this regard. They can also have a role in providing evidence of the efficacy of involving pharmacists in vaccine uptake; this may be especially convincing for countries where other medical professions who currently provide vaccination are reluctant to see other professional groups “compete” to provide immunization services. By supporting calls from within each country to improve vaccine access via pharmacy immunizations, international professional societies and organizations can provide additional impetus for change. In this regard, the EICA has a key role to play in providing support for the development of synergistic efforts and helping to coordinate educative initiatives across countries.

## Conclusions

Several key messages for the pharmacy profession came out of this stakeholder’s meeting. Firstly, it is important to ensure that the public better understands the need for vaccination across the lifespan, and trust-building interventions are critical to promote information acceptance. Second, payers, namely governments, need to understand the need, and provide the necessary resources to fund the service. In this context, pharmacists are health professionals who have the skills, capabilities and logistic capacity to vaccinate. There is a compelling need to promote the role of pharmacists as a key part of healthcare, prevention, and well-being promotion, working in concert with other health professionals towards a common goal, namely the care pathway to keeping patients healthy.

## Data Availability

Data are made available on request to the corresponding author.

## Abbreviations

EICA: European Interdisciplinary Council for Ageing
FIP: Fédération Internationale Pharmaceutique / International Pharmaceutical Federation
Flu: Influenza
GP: General practitioner
PSI: Pharmaceutical Society of Ireland
UK: United Kingdom

## References

[1] Demicheli V, Jefferson T, Ferroni E, Rivetti A, Di Pietrantonj C (2018). Vaccines for preventing influenza in healthy adults. Cochrane Database Syst Rev.

[2] Torres A, Peetermans WE, Viegi G, Blasi F (2013). Risk factors for community-acquired pneumonia in adults in Europe: a literature review. Thorax.

[3] Kimmel SR, Burns IT, Wolfe RM, Zimmerman RK (2007). Addressing immunization barriers, benefits, and risks. J Fam Pract.

[4] Determann D, de Bekker-Grob EW, French J, Voeten HA, Richardus JH, Das E (2016). Future pandemics and vaccination: public opinion and attitudes across three European countries. Vaccine.

[5] Schmitt HJ, Booy R, Weil-Olivier C, Van Damme P, Cohen R, Peltola H (2003). Child vaccination policies in Europe: a report from the summits of independent European vaccination experts. Lancet Infect Dis.

[6] Mills E, Jadad AR, Ross C, Wilson K (2005). Systematic review of qualitative studies exploring parental beliefs and attitudes toward childhood vaccination identifies common barriers to vaccination. J Clin Epidemiol.

[7] Jaca A, Mathebula L, Iweze A, Pienaar E (2018). Wiysonge CS.

[8] International Pharmaceutical Federation (FIP). An overview of current pharmacy impact on immunisation: A global report. The Hague, Netherlands: Fédération Internationale Pharmaceutique - FIP; 2016. Available at: https://fip.org/files/fip/publications/FIP_report_on_Immunisation.pdf (Access date: 19 June 2018).

[9] Pharmaceutical Society of Ireland. The Pharmaceutical Society of Ireland - Monthly statistics, March 2018. Available at: http://www.thepsi.ie/gns/Registration/public-registers/Statistics.aspx (Access date: 19 June 2018).

[10] Organization for Economic Cooperation and Development. Health at a Glance 2017: OECD Indicators. OECD Publishing, Paris. Available at: 10.1787/health_glance-2017-en. 2017.

[11] Statutory Instrument No. 525/2011 - Medicinal Products (Prescription and Control of Supply) (Amendment) Regulations 2011. Irish Statute Book 2011. Available at: http://www.irishstatutebook.ie/eli/2011/si/525/made/en/print (Access date: 19 June 2018).

[12] Statutory Instrument No. 449/2015 - Medicinal Products (Prescription and Control of Supply) (Amendment) (No. 2) Regulations 2015. Irish Statute Book 2015. Available at: http://www.irishstatutebook.ie/eli/2015/si/449/made/en/print (Access date: 19 June 2018).

[13] Pharmaceutical Society of Ireland. Guidance on the Provision of Vaccination Services by Pharmacists in Retail Pharmacy Businesses, version 4 April 2016. Pharmaceutical Society of Ireland, Dublin, Ireland. Available at: http://www.thepsi.ie/Libraries/Folder_Pharmacy_Practice_Guidance/PPGF_02_8_Guidance_on_the_Provision_of_Vaccination_Services_by_Pharmacists_in_a_Retail_Pharmacy_Businesses.sflb.ashx (Access date: 6 May 2018). 2016.

[14] Pharmaceutical Society of Ireland. Patient Feedback on the Flu Vaccination Service Provided in Pharmacies. March 2016. Pharmaceutical Society of Ireland, Dublin, Ireland. Available at: http://www.thepsi.ie/Libraries/Pharmacy_Practice/Report_on_Patient_Feedback_on_the_Flu_Vaccination_Service_Provided_in_Pharmacies.sflb.ashx (Access date: 6 May 2018). 2016.

[15] The Commonwealth Fund. 2016 Commonwealth Fund International Health Policy Survey of Adults. The Commonwealth Fund, New York, NY, USA. Available at: http://www.commonwealthfund.org/interactives-and-data/surveys/international-health-policy-surveys (Access date: 6 May 2018). 2016.

[16] Isenor JE, Alia TA, Killen JL, Billard BA, Halperin BA, Slayter KL (2016). Impact of pharmacists as immunizers on influenza vaccination coverage in Nova Scotia, Canada. Hum Vaccin Immunother.

[17] Buchan SA, Rosella LC, Finkelstein M, Juurlink D, Isenor J, Marra F (2017). Impact of pharmacist administration of influenza vaccines on uptake in Canada. CMAJ.

[18] Perman S, Kwiatkowska RM, Gjini A (2018). Do community pharmacists add value to routine immunization programmes? A review of the evidence from the UK. J Public Health (Oxf).

[19] Rai GK, Wood A (2018). Effectiveness of community pharmacies in improving seasonal influenza uptake-an evaluation using the Donabedian framework. J Public Health (Oxf).

[20] Atkins K, van Hoek AJ, Watson C, Baguelin M, Choga L, Patel A (2016). Seasonal influenza vaccination delivery through community pharmacists in England: evaluation of the London pilot. BMJ Open.

